# Exploring the needs and barriers for death education in China: Getting answers from heart transplant recipients' inner experience of death

**DOI:** 10.3389/fpubh.2023.1082979

**Published:** 2023-02-13

**Authors:** Wan Shu, QunFang Miao, JieHui Feng, GuanMian Liang, Jing Zhang, Jinsheng Zhang

**Affiliations:** ^1^School of Nursing, Hangzhou Normal University, Hangzhou, Zhejiang, China; ^2^School of Clinical Medicine, Hangzhou Normal University, Affiliated Hospital of Hangzhou Normal University, Hangzhou, China; ^3^Department of Cardiovascular Surgery, The First Affiliated Hospital of Medical College of Zhejiang University, Hangzhou, China; ^4^Zhejiang Cancer Hospital, Institute of Basic Medicine and Cancer (IBMC), Chinese Academy of Sciences, Hangzhou, Zhejiang, China

**Keywords:** heart transplantation, death attitude, near-death experience, death education, qualitative research

## Abstract

**Background:**

Promoting reflection about death may support better living, and how to carry out death education is an important issue to be addressed across the world. The purpose of the current study was to explore the attitude of heart transplant recipients toward death and their inner real experience to provide information for the development of death education strategies.

**Methods:**

A phenomenological qualitative study was conducted using a snowball method. A total of 11 patients who had undergone heart transplantation more than 1-year ago were recruited for the current study for semi-structured interviews.

**Results:**

A total of five themes were identified: “Not avoid talking about death,” “Feeling fear about the pain in the process of death”, “Wanting a good death at the end of life,” “The richness of feelings during near-death is surprising,” and “Being close to death makes people more receptive to death.”

**Conclusion:**

Heart transplant recipients have a positive attitude toward death and wish for “good death” at the end of life. These patients' near-death experiences and positive attitudes toward death during the course of their illness provided evidence of the need for death education in China and supported the experiential approach to death education.

## 1. Introduction

Death means the end of life (1). For each person, death is certain, while the time of death is uncertain. Death attitude refers to the response of people to their own death and that of others. Death attitude is an evaluative and stable internal psychological tendency exhibited by individuals in response to death, including both positive and negative attitudes (2). If we do not know and understand death correctly, our life will be shrouded in the shadow of death, and death will be a heavy burden that accompanies us all our life. Previous studies reported that establishing a positive and stable attitude toward death is an adaptation to the aging life process and guarantees high-quality social life (3).

In the early nineteenth century, the concept of “death science” gave rise to the development of death education in the United States. Death education refers to the various ways in which people are helped to gain knowledge about death and dying. It, thus, helps people in understanding the essence and significance of death and alleviates death anxiety and fear. The purpose of death education is to seek the meaning of life by focusing on the understanding of death (4). Several studies have extensively explored death education in Western countries, and death education is spread from primary college students to the public (5). Death education has gradually become a lifelong education for the public. In Asia, Iranian scholars found that the didactic method and death education based on the 8A model (alienation, avoidance, access, acknowledgment, action, acceptance, appreciation, and actualization) can effectively alleviate clinical nurses' death distress and promote their psychological health (6–8); scholars in Hong Kong, China, localized the 8A model and applied it to death education for social workers and achieved positive feedback (9). However, most Chinese people adopt an evasive attitude toward death owing to the influence of the Chinese traditional concept of life and death including the “taboo of death” (10). Research and promotion of death education in mainland China are relatively slow, and most studies focus on medical college students, medical personnel, dying patients, and other groups that are closer to death. Notably, the promotion of relevant theories and practices is still in the initial exploration stage (11). China has a large population, the latest statistics from the National Bureau of Statistics show that 9.93 million people died in 2018, accounting for one-fifth of the world's total death. Therefore, China urgently needs to develop death education for the general public, adapted to its national context. Under the cultural background that death is taboo in China, death education could be carried out by some special groups and gradually expanded to the general public.

For example, heart transplant recipients have unique features. Heart transplant recipients undergo a more complex emotional experience and have a more intuitive experience of facing death than other organ transplant recipients. While the heart is the initial power to preserve life in people, research shows that heart transplant recipients have to cope with the dual challenge of losing their own heart and accepting a donor's heart (12). Heart transplant is the most effective intervention for the treatment of end-stage heart disease, but some heart transplant recipients experience “psychological rejection”, meaning that they do not accept well the presence of another person's heart in their body (13). This experience of the body, mind, and spirit when facing death and near death is also known as a near-death experience. Studies involving patients with near-death experiences have shown that the particular experience of near-death has a direct impact on people's attitudes toward death (14). This qualitative study explored heart transplant recipients' experiences of illness and perceptions of life and death. The aim of the study was to gather information from the participants to guide the development of interventions to promote death education for the general public in China.

## 2. Materials and methods

### 2.1. Participants

A total of 11 patients who underwent heart transplantation were enrolled in this study in April 2022 through snowball sampling based on the saturation principle of qualitative research (15). As the incidence rate of anxiety and depression is highest in the year immediately after heart transplantation due to graft rejection and associated complications (12), only patients who submitted to heart transplantation more than a year ago were included in this study. Potential participants were only excluded if they did not agree to be involved in the study. Participants were assigned a code from Interviewee 1 to Interviewee 11. All participants in this study did not state any religion and had not previously been involved in any form of death education. General information on heart transplant recipients is shown in Table 1.

**Table 1:** General data for heart transplant recipients.

**Number**	**Age (old)**	**Gender**	**Educational status**	**Working status**	**Marital status**	**Etiology**
1	59	Male	Undergraduate	Retire	Married	Hypertrophic cardiomyopathy
2	32	Male	Junior high school degree	Unemployed	Married	Congenital heart disease
3	30	Male	Undergraduate	Incumbency	Married	Dilated cardiomyopathy
4	40	Female	Undergraduate	Incumbency	Married	Arrhythmogenic cardiomyopathy
5	31	Male	Undergraduate	Incumbency	Married	Dilated cardiomyopathy
6	41	Female	Junior high school degree	Unemployed	Married	Dilated cardiomyopathy
7	51	Male	Undergraduate	Incumbency	Married	Arrhythmogenic cardiomyopathy
8	30	Female	High school degree	Incumbency	Divorced	Inruptive cardiomyopathy
9	31	Male	Junior high school degree	Incumbency	Married	Dilated cardiomyopathy
10	32	Male	High school degree	Entrepreneurship	Married	Hypertrophic cardiomyopathy
11	58	Female	Technical secondary school degree	Retire	Married	Inruptive cardiomyopathy

### 2.2. Procedure

The current study adopted online telephony, guided by the phenomenological method owing to the challenges posed by the COVID-19 pandemic. We obtained the contact details of participants from the hospital and snowball the contact details of additional study subjects. Semi-structured in-depth interviews were conducted with participants based on a modified interview outline. The studies involving human participants were reviewed and approved by the Ethics Committee of Hangzhou Normal University (Number: 20190110). The final outline of the interview was discussed and revised with the heart transplant recipient, healthcare providers, and clinical psychologists as follows:

1. Has anyone ever discussed the topic of death with you?
2. What now comes to mind when you think of “death”?
3. Have you had a near-death experience? If so, did the experience make you believe differently about death?
4. Suppose you are at the end of your life, what kind of state or situation do you expect?
5. At the end of life, how do you want the medical staff and family to treat you?

The entire conversation was recorded during the interview, and the interview lasted for 40–60 min.

### 2.3. Measures

Audio recordings were transcribed into text within 24 h after the interviews and the text data were reviewed by another researcher. The interview data were analyzed using Nvivo 11.0 qualitative analysis software. Two authors (S.W and M.QF) analyzed and coded the data independently. Colaizzi's 7-step data analysis method was used for data analyses (16): (1) Read all the interview data several times and understand the interview data roughly; (2) Mark meaningful statements that are consistent with the research question; (3) Summarize and refine meaningful statements and code them; (4) Summarize the coded opinions, find common concepts, and form themes, subject groups, and categories; (5) Link the subject to the research object for detailed description; (6) Describe the essential structure that constitutes the phenomenon; and (7) Return the final analysis result to the research object for verification. This study met the criteria of Consolidated Criteria for Reporting Qualitative Studies (COREQ).

### 2.4. Quality control

The current study adopted the following measures to ensure the quality of the data: (1) Pre-interview was conducted to practice interview skills; (2) The researcher remained neutral throughout the interview session, only providing appropriate guidance and did not express personal views and opinions; and (3) Bracketing was achieved by writing a reflection diary during the analysis ^12^.

## 3. Results

### 3.1. Theme 1: Not avoid talking about death

Compared with healthy people, heart transplant recipients experience more sudden or near-death despair during the disease, and these experiences often make them unafraid to talk about death. Interviewee 5 said: “After this surgery, I accepted death very calmly. I am no longer afraid to talk about death. After all, I was once very close to it.” Interviewee 7 said: “I have discussed the topic of death with other people, but not very deeply. We have seen many things around us, including our own family members, parents, etc., so we have some insights about death.” Interviewee 10 said: “I sometimes wonder what the world will be like after I die, because after a major operation like a heart transplant. Death doesn't seem so scary.”

Furthermore, some respondents mentioned that although they do not avoid the topic of death, they are influenced by Chinese traditional concepts and do not actively mention the topic of death. Interviewee 11 said: “The traditional Chinese culture has prevailed for thousands of years, and the word ‘death' is relatively avoided.”

### 3.2. Theme 2: Feeling fear about pain in the process of death

Heart transplant recipients do not have a lot of fear of death. However, they are scared of the pain they may experience during the process of death. In the current study, Interviewee 2 said: “I'm terrified of experiencing the pain from the discomfort of my heart again. It is more acceptable to let me die a little bit more calmly.” Interviewee 6 said: “I am not afraid of death, but I am afraid of suffering pain.” Interviewee 10 said: “I am not afraid of death now, but I am fearful of the process of death. The process of death is terrible, is full of pain.”

### 3.3. Theme 3: Wanting to have a good death at the end of life

This study found that all 11 respondents expressed a reluctance to do meaningless resuscitation when referring to the end of life. This would reduce unnecessary waste of medical resources and also satisfy their desire to leave with less pain and hope for a good end. Interviewee 2 said: “(At the end of life) I think some treatments are unnecessary, it's a waste of money to do them, and then add a little more pain when I die, it's not necessary. I wish I could leave the world without any less painful.” Furthermore, Interviewee 5 said: “When I am in pain, I preferred administration of morphine and anesthesia to calm the pain. I want to be able to die without pain.” Interviewee 11 said: “I am very much in favor of euthanasia, so that it can not cause waste to the country, and individuals can leave with dignity.”

### 3.4. Theme 4: The richness of feelings during near-death is surprising

Nine of the 11 respondents in this study had experienced a near-death experience, and each person's near-death experience was different, but the experience was indeed diverse and rich. Interviewee 5 said: “I still remember that feeling, after I fell asleep, I was surrounded by a white patch, I felt a bright light all around me, and I was very sensitive to any sound. In retrospect, if I hadn't woken up, I might have just died.” Interviewee 6 said: “When I suddenly stood up, my eyes suddenly went black, and I thought to myself that the sky was still bright just now, but how did it get dark all of a sudden. It turned out to be the feeling of being on the verge of death, I was thinking it's over, my life is over, and then I fell to the ground.” Interviewee 7 said: “The near-death feeling was too scary, at that time I was already thinking a little confused, hallucinations. I heard the sounds of ghosts crying.”

### 3.5. Theme 5: Being close to death makes people more receptive to death

The findings of the current study showed that interviewees who had near-death experiences reported that close-to-death experiences enabled them to better understand and accept death. Interviewee 1 said: “In fact, when you are dying, you are not so afraid of death. Your brain is very calm.” Interviewee 6 said: “(Near-death experience makes me) more close to death. I don't think I will run away from death.”

In addition, one interviewee reported that reducing the fear of death in people is not always good. Interviewee 5 said: “If a technology developed for people can experience the state of being near-death. People would be less afraid of death, which would even lead to desire to commit suicide. I think in fact, people's fear of death, is not exactly a bad thing.”

## 4. Discussion

### 4.1. Unique illness experience brings more thoughts on death to heart transplant recipients

The attitude of heart transplant recipients toward death as well as their inner experience with death is unique. The development of individual death consciousness generally comprises five stages including “Discovery of Death”, “Fear of Death”, “Death Anxiety”, “Awe of Death”, and “Born to Die” (17). The findings of this study indicated that the personal experience of heart transplant recipients with death changed their attitude toward death from fear and anxiety to awe and acceptance of death. Death attitude is a complex subjective psychological variable, which changes constantly with time, changes in the surrounding environment, and major events encountered by oneself. Previous studies have revealed that sick people are more likely to accept death (17, 18), implying that physical illness is an important factor that can motivate people to think about death and the value of life. In addition, studies indicate that abstract cognition of death leads to death anxiety, whereas specific cognition of death leads to death reflection, which promotes acceptance of death (19). Therefore, the death experience in people improves their attitude toward death, which is in agreement with the findings of the present study. Some interviewees in the current study reported that they were not scared of talking about death, although they did not discuss death with others due to the influence of traditional Chinese culture. Preceding studies explored death attitudes (11) and reported that attention on death attitudes in China began approximately 20 years after other countries. The main reason is that due to traditional thinking, people regard death as a sensitive issue and are in a state of “death taboo”, which makes it difficult to implement relevant research and suggests the need to raise awareness and change the public perception about death in China to help the public form a healthy view of life and death.

### 4.2. Heart transplant recipients wish for a good death

Heart transplant recipients wish for a good and painless death. Painful near-death and sudden death experiences during the course of the disease make heart transplant recipients anxious about the pain and uncertainty of death. The 11 respondents in the current study reported that they were not willing to undergo meaningless invasive treatment including tracheotomy and endotracheal intubation at the end of life. The subjects preferred a painless and dignified death, which was in line with findings from other studies (20, 21). A good death saves patients, family members, and professional caregivers from pain and suffering, without violating clinical, cultural, and ethical standards (22).

The desire for a good death is challenging to achieve in China. A report on the quality of death index of 80 countries worldwide published in 2015 indicated that the quality of death of residents in mainland China ranks 71st globally (23). The quality of death of residents in mainland China is very low, which is mainly because hospice care in China is still in its infancy: the rate of hospice care in mainland China is only 1%. Medical resources in China are inadequate, hospice care wards and allied institutions are limited, and home-based care cannot be effectively implemented. Therefore, Chinese people's wishes for a good death cannot be effectively met. It has been found that excessive therapeutic interventions for end-stage patients increase the suffering of patients and may lead to massive wastage of medical resources (24). In the current study, one interviewee wished to undergo euthanasia. Euthanasia has been legalized in the Netherlands, 10 US jurisdictions, Switzerland (25, 26), and other countries. A portion of patients who are critically ill and incurable are willing to end their lives by euthanasia. However, the pertinent legislation on euthanasia in China is not clear.

Good death is consistent with the connotation of traditional Chinese culture (27). In China, “good death” is the fifth of the five blessings (28), and the first four blessings are longevity, wealth, health, and good morality. This study shows that heart transplant recipients have the wish for a good death, and this is consistent with studies involving oncology, and at the end of life, patients who wish for a good death (29), which suggests that to provide adequate person-centered care to society, we need to understand people's perception and personal wishes for end of life and promote the development of hospice care in accordance with Chinese conditions to meet the public's wish for a good death. Heart transplant recipients have more positive attitudes toward death because of their unique experiences and reflections on death. However, the health authorities should focus on the death education of the general public to change their attitudes toward death and help reduce the taboo about speaking about death and support people to better accept, deal, and cope with death.

### 4.3. Reflections on how to implement death education in China

This study found that heart transplant recipients have expectations of hospice care, and they want to achieve a good death. One of the difficulties is the lack of death education. Only when death education has reached a certain depth and breadth, and people have developed an understanding of death, will hospice care be most effective as a humanitarian form of care. Death education is, therefore, a necessary tool in the development of hospice care. There are currently some barriers to death education in China. First, in traditional Chinese culture, it is unlucky to talk about death, and as the interviewees in this study said, they rarely talk about death-related topics before they become ill due to traditional beliefs. Second, the specific form in which death education is conducted is also something that needs further consideration. One interviewee in this study, while mentioning the benefits of death education, stressed that a moderate fear of death is necessary, that being completely close to death and understanding it may drive suicide to occur—that fear of death is partly due to a lack of understanding of death. This suggests that we need to take this into account when doing death education for the general public and seek appropriate ways of educating about death for those who are prone to self-harm and self-injury, a part that remains to be explored further.

This study found that heart transplant patients were influenced by their experience of illness to think about the topic of death. These reflections played a role in the maintenance of their physical and mental health after surgery and in facing life positively—their attitude toward life and living it was not negative, despite experiencing major surgical trauma and even confronting uncertain postoperative complications and financial stress. This suggests that thinking about death is necessary to help people live better life (28). At this stage, death education in China is scarcely available due to people's concern that in some circumstances, death education may have a negative impact on people, for example, it could encourage people to suicide (30).

After clarifying the feasibility and necessity of death education, we need to consider the appropriate way of death education, which is currently carried out in China through a few education courses (31). From the experience of heart transplant recipients, the near-death experience is an educational process that brings the patient closer to death and understanding it. Previous studies reported that the formation of specific cognition of death helps in establishing a correct view of death and reduces strangeness, fear, and anxiety about death (19). Therefore, for the general public, experiencing death education is a more intuitive way of teaching about life and death. The results of this study indicated that heart transplant recipients are not afraid of death and tend to accept it calmly owing to the matchless near-death experience and illness experience. This death attitude promotes the recovery of transplant recipients' physical and mental health and helps them find meaning in life and promotes the desire for a good end-of-life. Therefore, the unique near-death experience of heart transplant recipients and their rich inner experience of death provide a basis for the development of experiential death education. Experiential education currently mainly focuses on visiting funeral homes, attending funeral services, and visiting the critically ill, thus improving knowledge of death. In the current study, 9 out of 11 respondents reported that they had experienced a near-death experience. The experience of confronting death made their descriptions of death more tangible and reduced their anxiety toward death. “Near-death experience” comprises physical, mental, and spiritual experiences that people undergo on the verge of death and is a close sensory experience with “death” (32). The findings of a previous study indicate that this experience can expand the breadth and depth of life and result in more thinking about the meaning of life (33). Shanghai, China previously had a public-oriented death experience pavilion named “wake up”, where people would undertake death rituals, including playing a dirge, putting on clothes, eating a farewell meal, leaving a suicide note, and sleeping in a coffin of the medium. However, the experience museum was permanently closed in April 2019 subject to morals and the limitation of traditional Chinese culture. This suggests that death education and experiential death education to be delivered in China need to be further explored.

Death education helps people acquire knowledge on death and dying and helps in cultivating and improving the ability of the public to cope and deal with death events (28). In addition, death education reduces the acquired fear of death in people, which is an important prerequisite for achieving a good death. It is also important to implement death education from a hospice perspective. First, in the dying state, death education can lead to a more rational allocation of medical resources. China is a country with a large population and reducing unnecessary medical measures can effectively reduce the pressure on healthcare (34). Second, at present, healthcare services in China are mainly focused on the treatment and care of patients, without sufficient attention to the bereavement care of family members. Previous studies indicated that experiencing death through near-death experiences or other ways and extending mourning care to people who have lost relatives and friends helps people carry out “After-Death Communication”(ADC) (35). These activities promote subjects to change from fear of death to peace as well as from anxiety of death to peace. These findings indicate that near-death experience is a means to understand death, and this experience or other experiential forms should be added to death education in future to broaden the form of death education.

## 5. Limitations

The recruitment method through snowball is likely to have contributed to selection bias. Moreover, this finding most likely represents the experience of heart transplant recipients and not of the general population. It is still necessary to be cautious to extend the conclusions of this study to public death education. In addition, one's religious beliefs are closely related to one's attitudes toward death, and none of the subjects in this study had religious beliefs. Further research is needed to explore how religious heart transplant recipients view death and to clarify the possible differences.

## 6. Conclusion

This study adopts a phenomenological research method to conduct an in-depth analysis of the death perception and attitudes of 11 heart transplant recipients, which shows that heart transplant recipients present a non-avoidant attitude toward death; they wish for a pain-free and peaceful death, and health services should further explore ways to address these wishes. More importantly, this study provided evidence about the need for death education in China and supports the experiential approach to death education.

## Data availability statement

The raw data supporting the conclusions of this article will be made available by the authors, without undue reservation.

## Ethics statement

The studies involving human participants were reviewed and approved by Ethics Committee of Hangzhou Normal University (Number: 20190110). The patients/participants provided their written informed consent to participate in this study. Written informed consent was obtained from the individual(s) for the publication of any potentially identifiable images or data included in this article.

## Author contributions

Conceptualization: WS and QM. Methodology, formal analysis, investigation, and writing—original draft preparation: WS. Software, supervision, and project administration: QM. Validation: QM, JF, and GL. Resources: GL. Data curation: JinsZ. Writing—review and editing: WS and JingZ. Visualization: JF. All authors have read and agreed to the published version of the manuscript.
